# Fentanyl Exposure and Detection Strategies Utilized by Clinical Trial Participants Seeking Linkage to Opioid Use Disorder Treatment at a Syringe Service Program

**DOI:** 10.1007/s13181-023-00979-7

**Published:** 2023-12-04

**Authors:** Dennis P. Watson, Bradley Ray, Peter Phalen, Sarah E. Duhart Clarke, Lisa Taylor, James Swartz, Nicole Gastala

**Affiliations:** 1 Chestnut Health Systems, Lighthouse Institute, 221 W Walton Street, Chicago, IL 60610 USA; 2 RTI International, 3040 Cornwallis Road, Research Triangle Park, Chapel Hill, NC 27709 USA; 3 Department of Psychiatry, University of Maryland School of Medicine, 655 W Baltimore Street, Baltimore, MD 21201 USA; 4 Jane Addams College of Social Work, The University of Illinois at Chicago, 1040 W Harrison Street, Chicago, IL 60607 USA; 5 Department of Family Medicine, Mile Square Health Centers, University of Illinois College of Medicine at Chicago, Chicago, IL USA

**Keywords:** Harm reduction, Overdose, Fentanyl, Opioid use disorder, Overdose disparities

## Abstract

**Introduction:**

The USA continues to face a fentanyl-driven overdose epidemic. Prior research has demonstrated users of illicit opioids are concerned about fentanyl exposure and overdose, but the strategies they report using to detect fentanyl’s presence lack empirical support. This study compares self-report and biologically detected fentanyl use and investigates overdose risk and risk reduction behaviors among a sample of high-risk people who use opioids.

**Methods:**

Structured enrollment interviews conducted as part of a larger clinical trial assessed self-reported fentanyl exposure as well as strategies used to determine believed fentanyl exposure and prevent overdose among 240 participants enrolled at a Chicago, IL syringe service program. Urinalysis measured actual fentanyl exposure.

**Results:**

Most participants identified as African American (66.7%) and had considerable overdose experience (76.7% lifetime and 48% in the past year). Most also tested positive for fentanyl (93.75%) despite reporting no past year use of fentanyl or fentanyl-adulterated drugs (64.17%). The most utilized approaches reported for identifying fentanyl exposure were stronger effects of the drug (60.7%), sight or taste (46.9%), and being told by someone using the same drugs (34.2%). Few participants (14%) reported using fentanyl test strips. No significant associations were identified between self-report and urinalysis measures or urinalysis results and risk reduction strategies.

**Conclusion:**

This study adds to prior fentanyl exposure risk research. The disconnect between participants’ fentanyl detection methods and reported overdose experiences supports the need for more research to identify and understand factors driving access and use of overdose prevention resources and strategies.

## Introduction

Approximately half of the world’s opioid-related overdose deaths occur in the USA [1, 2]. These deaths are currently driven by fentanyl saturation in the illicit drug market [3, 4]. Fentanyl is easier to produce than heroin and can be smuggled in small batches due to its potency [5]. Given the clandestine nature of the illicit drug market, it is difficult to surveille trends; however, research suggests the first US-based illicit fentanyl laboratory was discovered in Kansas in the 1990s. Fentanyl subsequently appeared periodically in heroin batches until the mid-2000s when there was a sharp increase from the Midwest to the Northeast corridor, resulting in the national spread of fentanyl during the 2010s [6]. Fentanyl’s saturation of the North American drug supply expanded rapidly during the COVID-19 pandemic, and it is now the primary driver of US opioid overdoses [7–9]. While prior research has demonstrated users of illicit opioids are concerned about fentanyl and attempt to avoid its exposure [10–12], the accuracy of the documented strategies used by this population to detect fentanyl and prevent overdose has not been adequately investigated.

While people who use opioids may attempt to avoid fentanyl because of the higher risk of overdose [13] and test strips are available to detect fentanyl, many users instead report relying on personal detection methods such as sight, taste, smell, and the resulting high experienced after dosing [14, 15]. For example, individuals who use opioids have reported the presence of fentanyl can be identified by a powdery texture, abnormal colors like gray or purple, a sweeter taste, or sensations associated with use such as heavier sedation or “pins and needles” [14]. However, the accuracy of such detection methods is unknown and, even when these strategies are employed, research has found users of opioids’ beliefs regarding fentanyl use are inaccurate [16–21]. These studies rely on various drug testing methods (urine or saliva drug screening) with prevalence varying by sample and time frame. One North American study reported more than 50% of participants who denied use tested positive for fentanyl [20]; however, this work was completed prior to the COVID-19 pandemic’s onset. Further saturation of fentanyl within the street drug supply and associated changes in knowledge and expectations among people who use opioids require more current investigation in this area.

Prior research in this area has demonstrated considerable discrepancies between client expected and actual fentanyl exposure as verified by biological testing [22–26]. However, no US-based studies to date have investigated these factors when overdoses were rising considerably during the COVID-19 pandemic [9] nor among a population of individuals seeking services through a syringe service program (SSP), a harm reduction setting designed to support people who use opioids and where participants are likely to feel less stigma about reporting fentanyl use [27–29]. The current study sought to fill this research gap by comparing self-reported and biologically detected fentanyl use and investigating overdose risk and fentanyl detection strategies among a sample recruited at an SSP in Chicago, Illinois during the COVID-19 pandemic.

## Methods

Data were collected as part of the STAMINA (Syringe Service Telemedicine Access for Medication-Assisted Intervention through NAvigation) study (ClinicalTrials.gov ID: NCT04575324), a randomized clinical trial testing a telemedicine treatment linkage intervention. A description of the full STAMINA trial procedures is published elsewhere [30]; however, all information relevant to the design, recruitment, measures, and procedures relevant to the current analysis are described below.

### Participant Recruitment

Participants were enrolled in the larger trial between August 24, 2020, and June 30, 2022. All individuals were informed of the opportunity to participate in the study at the SSP site, through community outreach workers, or by encountering offsite recruitment materials. In addition to providing clean syringes, this site offers a range of harm reduction services (e.g., naloxone and condom distribution, safer drug use kits, and HIV and HCV testing), making it a resource for people who use drugs whether they inject or not. To be eligible for the trial, participants had to (a) be at least 18 years of age; (b) speak English; (c) meet clinical criteria for a past-year opioid use disorder of any severity level [31]; (d) express interest in receiving medication to treat opioid use disorder (MOUD, e.g., methadone, buprenorphine, or injectable, long-acting naltrexone); and (e) reside in Cook County, Illinois (to increase likelihood of trial follow-up interview completion). Individuals were excluded if they were (a) planning to move outside of Cook County within the next 6 months (to help ensure completion of clinical trial follow-up procedures), (b) under criminal justice supervision that required serving a jail or prison sentence within 6 months, (c) experiencing severe withdrawal symptoms (indicating a likely need for immediate intervention) [32], (d) currently taking any prescription MOUD, or (e) demonstrating inadequate ability to provide informed consent.

### Procedures and Measures

All data reported in the current study are from in-person structured interviews and urine testing completed by a research assistant during participant enrollment. Measures from the interviews include client sociodemographic characteristics (current age, age at first opioid use, gender identity, race, ethnicity, income, and lifetime injection drug use). Participants were also asked about their current preference for using either heroin or fentanyl and to report a count of lifetime overdoses and time since the last overdose (i.e., an opioid poisoning event resulting in the need for another person to take a life-saving action such as administering naloxone or CPR or calling emergency medical services). The following questions were asked to assess perceived fentanyl exposure and application of overdose prevention/harm reduction strategies: “In the past year, have you taken other opioids such as fentanyl or carfentanil either alone or in combination with heroin or another drug?” (yes/no); [if yes] “How did you determine fentanyl was present?” (see Table 4 for response categories); “In the past 3 days, did you intentionally use fentanyl or other forms of a synthetic opioid such as carfentanil either alone or in combination with another drug such as heroin?” (yes/no); “In the past year, have you taken any of these precautions to make an overdose less likely?” (see Table 4 for response categories). Participants completed a urine drug test at the time of their interview using a T-Cup® CDOA-9165EFTK 16-panel compact instant drug test cup (manufactured by Wandfo Biotech), which the manufacturer’s insert states is for forensic purposes and provides up to 99% accuracy and detects nor-fentanyl, a fentanyl metabolite, with a maximum detection time of 3 days and a cutoff level of 20 ng/mL. The test also assessed the presence of other drug metabolites. Interviews took approximately 30–45 min to complete, and all participants received a $35 incentive. All human subjects procedures were reviewed and approved by the Institutional Review Board of Chestnut Health Systems.

### Analysis

Participants recruited before May 3, 2021, were excluded from the current analysis because a saliva-based screening was used for fentanyl detection between these dates (the change in tests was a result of supply issues related to the pandemic). Descriptive statistics were calculated for all measures. Chi-square tests were used to compare perceived past-year and past 3-day fentanyl use with urine test results and to assess relationships between urinalysis results and overdose prevention and fentanyl detection strategies. All statistics were calculated using R V.4.2.0 [33].

## Results

**Fig. 1:**
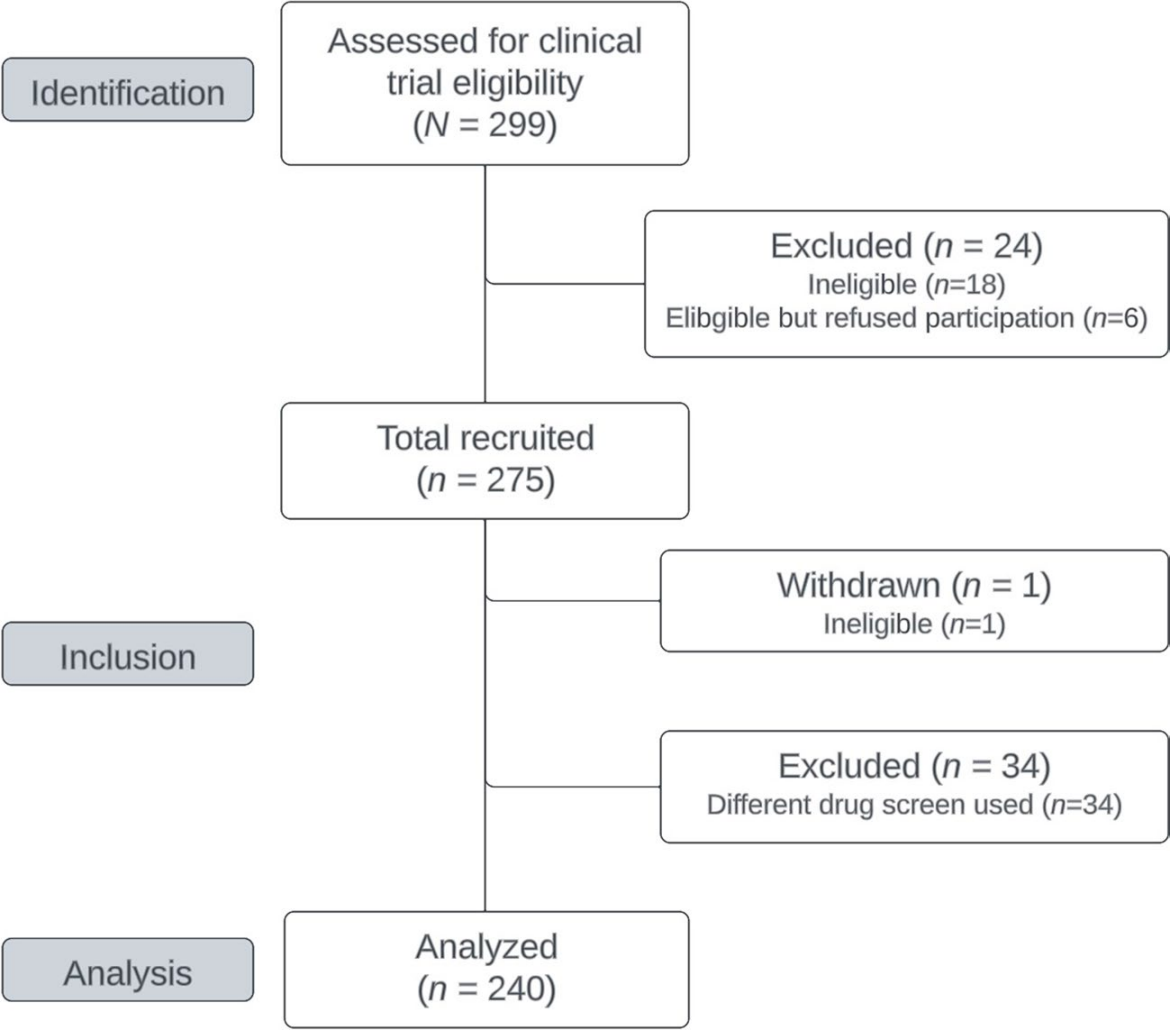
Participant flow diagram

Figure 1 presents the flow of participants and their data through the study procedures. The total screened for trial eligibility was 299, of which 24 were excluded. Of the 275 who met eligibility for the larger clinical trial, one was found to be ineligible after starting data collection and withdrawn. Another 35 were excluded from the current analysis because they completed a saliva vs. urine drug screen. The final analytical sample was 240.

### Sample Characteristics

The majority of participants were male (77.9%), African American (66.7%), and averaged 50 years of age (Table 1). Approximately two-thirds (65.4%) reported an income of under $10,000 per year. The average reported age of opioid use initiation was 22.7; less than half (41.7%) reported any lifetime injection drug use. There was a median of 24.5 reported lifetime overdoses with 48% indicating an overdose occurring within the past year.

**Table 1:** Participant demographic characteristics

	Median	IQR
Age at interview	50	39.6–55.7
Age at first opioid use	19	17–26
Gender identity	*n*	%
*Male*	187	77.9%
*Female*	52	21.7%
*Transgender*	1	0.4%
Race categories^a^	*n*	%
*African American*	160	66.7%
*White*	51	21.3%
*American Indian*	5	2.1%
*Asian*	1	0.4%
*Other*	35	14.6%
Ethnicity		
*Latino/Hispanic*	41	17.1%
*Not Latino-Hispanic*	199	82.9%
Income	*n*	%
*$0–$9,999*	157	65.4%
*$10,000–$19,999*	47	19.6%
*$20,000–$29,999*	19	7.9%
*$30,000–$100,000 or more*	14	5.8%
*Don’t know*	3	1.3%
Lifetime injection drug use	*n*	%
*Yes*	100	41.7%
*No*	140	58.3%
Lifetime overdoses	Median	IQR
	24.5	1–510.6
Time since last overdose	*n*	%
*Past month*	39	16.3
*Past 6 months but more than 1 month ago*	50	20.8
*Past year but more than 6 months ago*	27	11.3
*More than 1 year ago*	64	26.7
*Never overdosed*	56	23.3
*Don’t know/refused*	4	1.6

^a ^Cells may total >100% as participants were invited to select more than one option

### Opioid Use, Fentanyl Exposure, and Fentanyl Detection Strategies

Table 2 displays self-report fentanyl use and urine detection results. Most participants (98.75%) identified heroin as the drug they had the strongest urgency or craving to use, with only three participants (1.25%) endorsing fentanyl. Most reported having used opioids within the past 24 h (91.67%) and one-third (34.17%) reported fentanyl use in the past year. Despite this, most participants (93.75%) had positive urinalysis results. Urine test results also demonstrate high rates of detection of oxycodone (80.7%), other opioids as measured by the presence of the metabolite morphine (73.9%), stimulants (60.7%), and other drugs (93.7%) in combination with fentanyl.

**Table 2:** Self-reported fentanyl use and urine detection

	*n*	%
Opioid of preference		
* Heroin*	237	98.75
* Fentanyl*	3	1.25
When is the last time you used any opioid?	*n*	%
* Within the past 24 h (1 day)*	220	91.67
* Within the past 48 h (2 days)*	11	4.58
* Within the past 72 h (3 days)*	0	0.00
* Within the past week (7 days)*	6	2.50
* Within the past month (30 days)*	1	0.42
* More than a month ago (*> *30 days)*	1	0.42
* Don’t know/refused*	1	0.42
Perceived past year fentanyl exposure	*n*	%
* No*	154	64.17
* Yes*	82	34.17
* Unknown or not sure*	3	1.25
* Missing*	1	0.42
Fentanyl urinalysis test results	*n*	%
* Positive*	225	93.75
* Negative*	15	6.25
Positive polysubstance urinalysis test results*	*n*	%
* Norfentanyl* + *oxycodone*	151	80.70
* Norfenanyl* + *opioids*	102	73.90
* Norfentanyl* + *stimulant*	145	60.70
* Norfentanyl* + *other non-opioid non-stimulant drug*	224	93.70

*Categories total to > 100% because categories are not mutually exclusive

### Comparison of Self-Report Fentanyl Use and Biological Testing

Table 3 compares participants’ self-reported beliefs about the use of fentanyl with urinalysis results and shows 93.8% of participants tested positive for fentanyl indicating they had used fentanyl recently, despite most participants (86.3%) stating they had not intentionally used fentanyl within the self-report window. Participants’ intentional use of fentanyl within the past 3 days did not have a statistically significant association with actual fentanyl use, as participants who tested negative were as likely to report intentional use as participants who tested positive. Chi-squared tests demonstrate that beliefs about past-year fentanyl use (whether intentional or unintentional) also had no significant association with testing positive for fentanyl.

**Table 3:** Comparison of urinalysis results and self-reported fentanyl use and detection

	*Urinalysis fentanyl results*		*Χ*^2^	*p*
Perceived past year fentanyl exposure (intentional or unintentional)*	Positive	Negative	1.50	0.27
* Yes*	79	3		
* No*	145	12		
Perceived past 3-day fentanyl exposure (intentional)	Positive	Negative	0.00	1.00
* Yes*	13	2		
* No*	194	31		

^*^Numbers add to 239 rather than 240 because self-reported exposure for one participant was missing

### Association Between Urinalysis Results and Overdose Risk Reduction and Fentanyl Detection Strategies

Table 4 shows the most endorsed precautions to reduce overdose were (1) taking a smaller amount of the drug than usual (57.9%), (2) taking a smaller test dose of the drug first (52.1%), (3) buying from a trusted dealer (36.7%), (4) making sure naloxone was available (33.3%), and (5) using with someone else present (30.4%). The table also shows considerable variety in self-reported methods of fentanyl detection, with the majority (60.7%) stating they believed fentanyl was present because the effect of the drug was stronger than expected. A small minority (14%) had used fentanyl test strips and only 16.5% had intentionally sought out drugs containing fentanyl. Chi-square test results demonstrate no relationship between positive fentanyl urinalysis results and either overdose risk reduction or fentanyl detection strategies.

**Table 4:** Associations between positive fentanyl urinalysis results and overdose risk reduction and fentanyl detection strategies

	*n*	%	Percent positive fentanyl urinalysis (95% CI)	*X*^2^	*p*
Past year precautions taken to reduce overdose risk^a^					
* Used a smaller amount of the drug than usual*	139	57.9	95.7 (90.4–98.2)	1.39	0.24
* Taken a small test dose of the drug first*	125	52.1	96.8 (91.5–99)	3.13	0.07
* Buying my drugs from a dealer I trusted*	88	36.7	96.6 (89.7–99.1)	1.23	0.27
* Made sure naloxone was available before using*	80	33.3	96.3 (88.7–99.0)	0.72	0.39
* Tried to use with someone else present*	73	30.4	97.3 (89.6–97.3)	1.43	0.23
* Visual inspection or tasting it first*	67	27.9	97.0 (88.7–99.5)	1.01	0.32
* Tested the drug for fentanyl using a test strip*	28	11.7	96.4 (79.8–99.8)	0.04	0.84
* No, I have not taken any of these precautions*	19	7.9	94.7 (71.9–99.7)	0.0	1
* Don’t know/refused*	1	0.4	0.0	NA	NA
Reported fentanyl detection strategies (*n* = 79)^a,b^					
* The effect of taking the drug was much stronger than I expected*	48	60.7	95.8 (84.6–99.3)	0.00	1
* I could tell by looking at or tasting the drug before I used it*	37	46.9	97.3 (84.2–99.9)	0.00	1
* Another user told me fentanyl was present*	27	34.2	88.9 (69.7–97.1)	3.35	0.06
* Same supplier and they always contained or were mostly fentanyl*	24	30.4	95.8 (76.9–99.8)	†	†
* I purposely sought out fentanyl or drugs that contain fentanyl*	13	16.5	92.3 (62.1–99.6)	0.00	0.992
* Some other way*	12	15.2	100 (69.9–100.0)	†	†
* I tested the drug before I used it with a test strip*	11	14.0	90.9 (57.1–99.5)	†	†
* My dealer told me it contained fentanyl before I bought it*	8	10.2	87.5 (46.7–99.3)	†	†
* Don’t know/refused/other*	1	1.3	100 (5.5–100)	†	†

^a^Cells may total > 100% as participants were invited to select more than one option

^b^Participants were only asked fentanyl detection strategies if they reported fentanyl use; two-sample tests of equal proportions were used to assess whether there was a relationship between each precautionary strategy and positive urinalysis results

^†^Expected values of one or more cells is less than 5 and chi-squared approximation may be incorrect

## Discussion

This study compared self-reported fentanyl use collected through structured interviews to fentanyl urinalysis results among a sample of SSP clients who entered a clinical trial testing a telemedicine opioid use disorder (OUD) treatment linkage intervention. The sample was predominantly African American with a recent and frequent history of nonfatal overdose, who primarily sought heroin and wanted to avoid fentanyl and prevent related overdose. Despite only three participants stating they prefer fentanyl, more than one-third believed they had used it with the most common method of fentanyl detection reported being having experienced more intense effects after use. The most frequently endorsed overdose prevention strategies were to take a small test dose and buy from a familiar/trusted source. While most participants stated they had not been exposed to fentanyl in the past year, urinalysis demonstrated a large majority had done so within the past 3 days, with no meaningful observed variability in the use of overdose prevention or fentanyl detection strategies and urinalysis results.

The most popular detection method of a more intense experience (or high) aligns with previous studies investigating people’s ability to detect fentanyl in their drugs. For instance, Duhart Clarke et al. [14] found that people in North Carolina reported first recognizing the saturation of fentanyl in the illicit opioid market by changes in physical sensations experienced when using. Fentanyl was reported as being much more sedative, having a stronger initial “rush” but less duration, and being accompanied by novel sensations such as pins and needles, itchiness, and chest tightening [34]. Further, these prior studies have described users of opioids learning how to discern fentanyl over time by first identifying these physical sensations and changes in their high, connecting the changes to differences in a drug’s appearance, and confirming fentanyl’s presence with tools such as fentanyl test strips [14, 34]. In the current study, most participants reported no knowledge of the fentanyl exposure indicated by their urinalysis despite using a variety of methods to detect fentanyl; in fact, the use of fentanyl test strips was one of the least used methods. This highlights the need for efforts to educate users of opioids that fentanyl adulteration should be assumed and to expand community access to drug-checking resources. When combined, such efforts could encourage more people who use opioids to take necessary overdose prevention precautions (e.g., not using alone and carrying naloxone) [35]. Spectrometry apparatuses can provide more accurate information on drug potency and are feasible to implement in community settings such as SSPs [36]. However, this approach is recommended to be implemented in combination with fentanyl test strips, which are more sensitive to fentanyl detection [37]. Test strip distribution also has a wider reach than spectrometry methods since they can be carried by individuals and used in any location. Even if a positive test strip result does not change opioid consumption, recent research suggests test strip use is associated with carrying naloxone [38], which could help prevent fatal overdoses. That said, test strips do carry some risks in states with laws designating them as paraphernalia, and individuals should be educated about these laws as part of test trip distribution [39].

It is possible that self-stigma, driven by fear-based messaging and misinformation about fentanyl, may have inhibited participants from disclosing their fentanyl use, creating the current study’s discrepancy between participants’ self-reported fentanyl use and urinalysis results [40]. While this may have played a role, a more likely scenario is that participants were unaware of their recent fentanyl use. This is because prior research has demonstrated people who use opioids feel less stigmatized and are more comfortable in SSP settings [28, 29], and there is demonstrated validity of self-report drug use when accompanied with drug test measures, such as urinalysis [41–43]. Furthermore, nearly all participants openly reported using opioids in the past 24 h, reported a preference for heroin, and were taking precautions in an attempt to prevent overdose due to drug adulteration. This demonstrates a willingness to use overdose prevention strategies that can be leveraged to encourage both increased use of fentanyl test strips and other effective drug testing technologies.

Most participants in this study identified as African American, which has potential implications for results related to risk perceptions and precautions. There is evidence that African Americans are more likely to insufflate/snort than inject drugs as compared to Whites [44, 45]. This is also suggested in our data, as the majority of participants indicated they had never injected drugs. Among those who use opioids, it is often believed that snorting carries less overdose risk [14, 46], which may influence the use of risk reduction strategies—that is, perceptions of reduced risk may result in the use of less effective overdose prevention strategies (e.g., relying on visual inspection vs. using a fentanyl test strip). Future studies should seek to identify and understand race-based differences in substance use and their impact on risk behaviors. Most participants’ urine tested positive for recent fentanyl use, despite nearly all participants reporting a preference for heroin and taking precautions to reduce overdose risk, including inspecting their drugs for adulteration. This demonstrates how fentanyl’s saturation of the illicit opioid market has diminished people’s ability to avoid using fentanyl. It also demonstrates the heightened need for public health strategies and treatment models that incorporate harm reduction, including low-barrier MOUD [47, 48]. Policies guiding such strategies must consider differences in culture and drug use patterns to ensure appropriate access and uptake by minority populations if they are going to appropriately address growing health disparities between subpopulations of people who use opioids.

There are limitations to consider when interpreting these study results. The sample reflects people who use opioids seeking treatment through a Chicago-based SSP and might not be fully generalizable to other service and geographic settings. However, prior research in this area has been largely regionally focused, and our results provide some insights regarding fentanyl-related attitudes, use, and avoidance behaviors among a majority African American sample, which is an understudied group. For example, we are aware of only one other study [25] that compared self-report fentanyl use with biological test results among a sample of participants from Maryland, the majority of which were African Americans who mostly (76%) did not inject. While most of their sample indicated trying to avoid fentanyl, they did not probe specifically into the utilization of overdose prevention strategies. Another limitation to generalizability already pointed to above is that the saturation of fentanyl within the street drug supply coincided with the pandemic’s onset. At this point, expectations related to fentanyl adulteration among people who use opioids have likely changed, and these might have affected associated risk and risk reduction behaviors.

Other limitations of this study are related to specific questions and the biological test used. We were unable to assess the sensitivity and specificity of self-report/detection strategies due to interview questions not specifically asking about strategy use within the urine test’s 3-day detection window. Likewise, we asked about general rather than time-specific use of risk reduction strategies, and this could have negatively impacted our ability to detect an observed relationship between any of these strategies and urine test results. Future research should seek to determine the time since suspected fentanyl use and the use of risk reduction strategies to improve the accuracy of resulting findings. Relatedly, our question about perceived past 3-day fentanyl exposure asked specifically about intentional use. While our question covering past-year use does include this 3-day window, it does not allow us to understand possible recent unintentional exposure among the majority of the 79 participants who reported they had taken fentanyl in the past year. Confirmatory lab testing was not used in this study because it was not required clinically for the larger trial and there was inadequate storage room at the SSP locations. While fentanyl has a short half-life (3–12 h) and only a small amount is secreted in urine, the manufacturer of the test used in this study states it is reactive to fentanyl’s metabolite norfentanyl for a maximum 3-day urine detection window. Finally, it is possible that polysubstance use and the possible presence of other adulterating substances (such as nitazines and xylazine) could have affected urine test results through cross-reactivity or metabolic interactions [49–51].

## Conclusions

The fact that most participants tested positive for fentanyl use despite believing they were using fentanyl-free heroin reinforces the reality that people who use illicit opioids cannot avoid fentanyl and public health professionals must consider this when designing appropriate overdose reduction strategies. The lack of any significant relationship between detection methods and actual exposure underscores the need for more policies and resources in the USA to support community drug-checking services and low-barrier MOUD access, as well as the investigation into the potential benefits of safe-supply strategies (i.e., making safer opioids available to those at high risk of overdose through off-label prescribing of pharmaceutical-grade opioids) [52, 53]. While generalizability is limited by geography and a rapidly changing drug supply [54], the majority of African-American representation in our sample provides insights regarding fentanyl exposure among a minority population at high risk for opioid overdose relative to the larger and primarily White population of people who use opioids and who are better represented in the literature [55–57]. Our study supports the growing need for harm-reduction interventions that can effectively help people avoid using contaminated drugs or help them to reduce the risks of using contaminated drugs should they choose to do so.

## Data Availability

De-identified data will be made available upon request.
